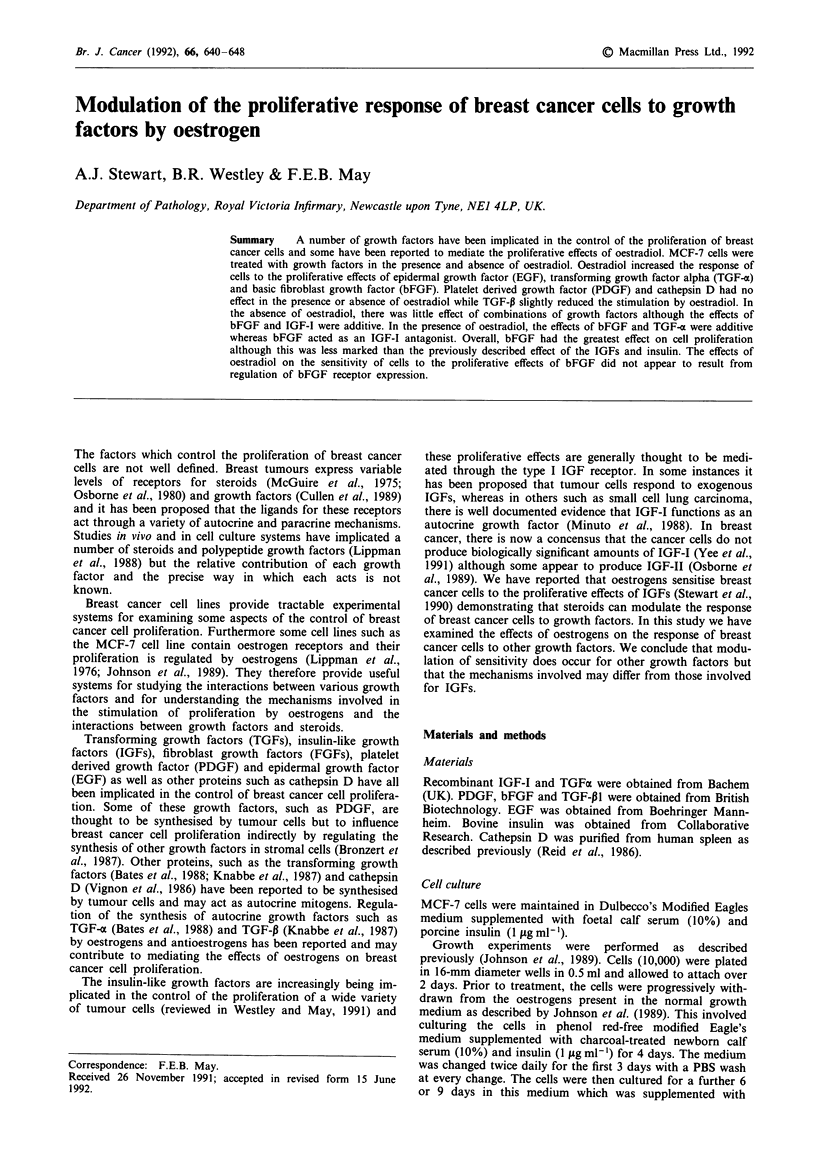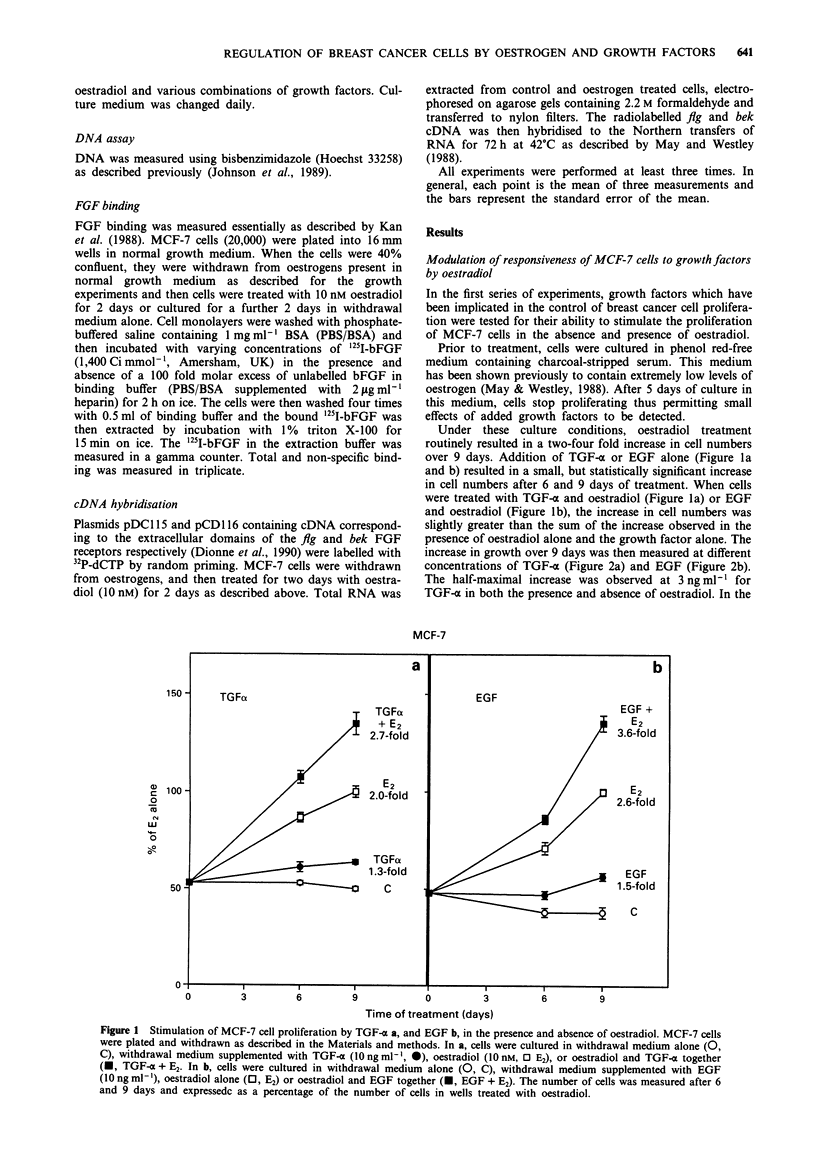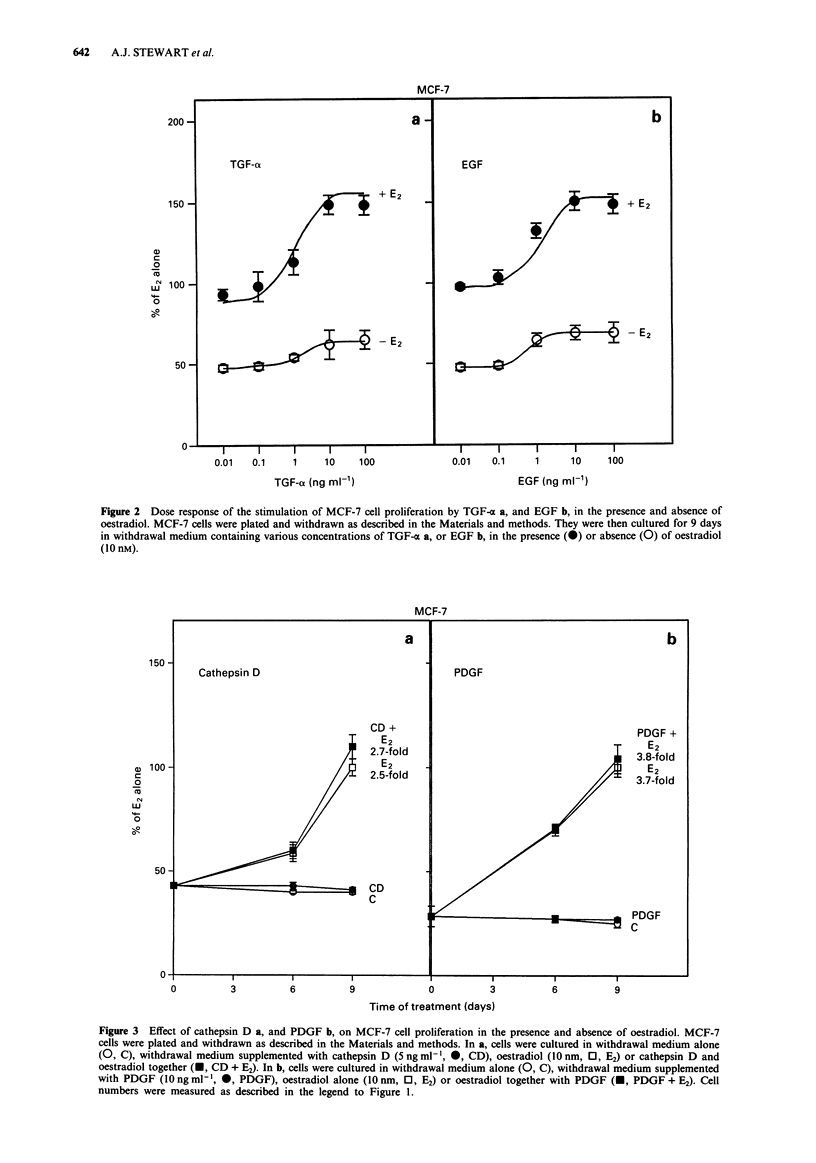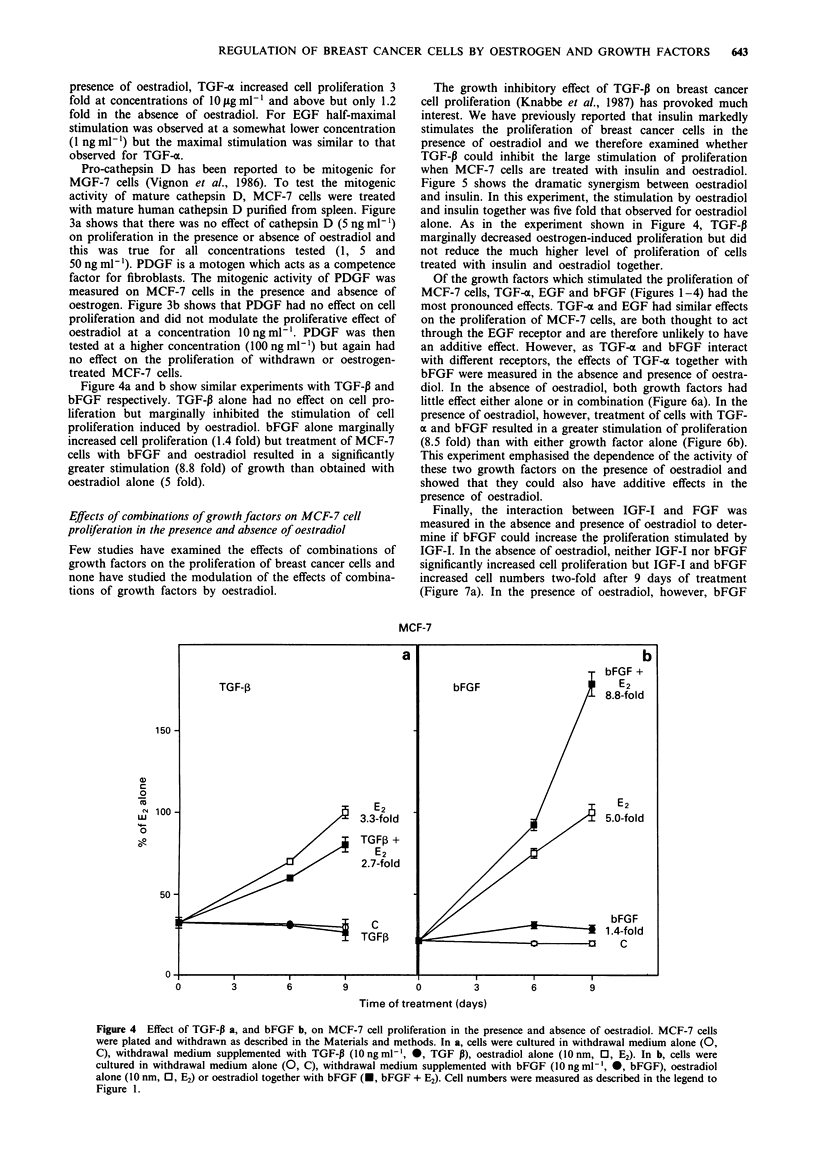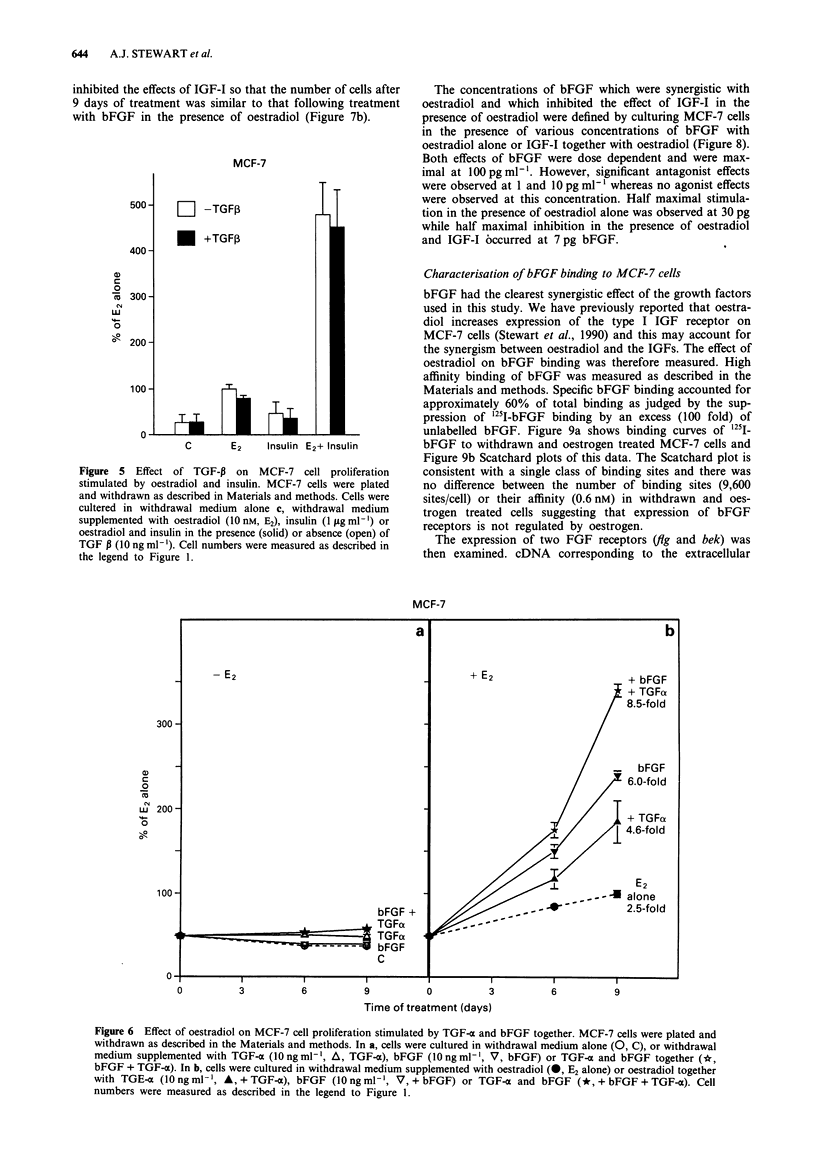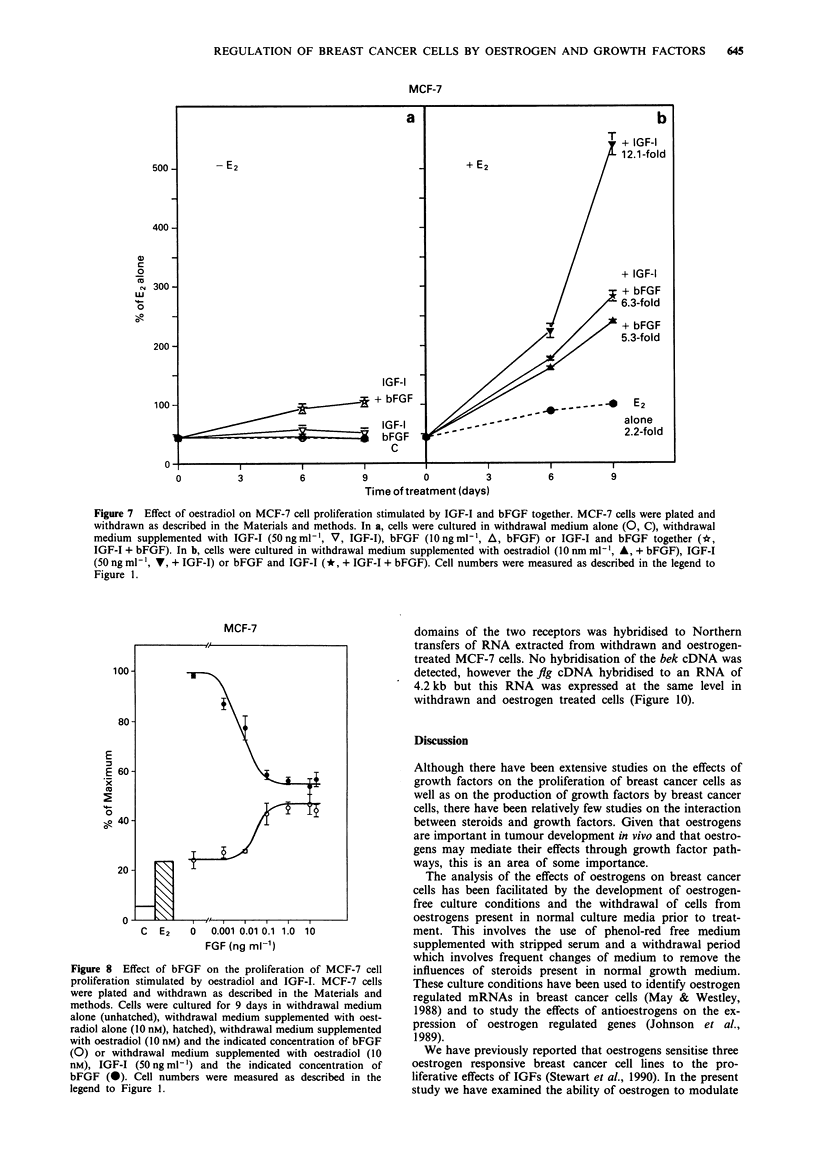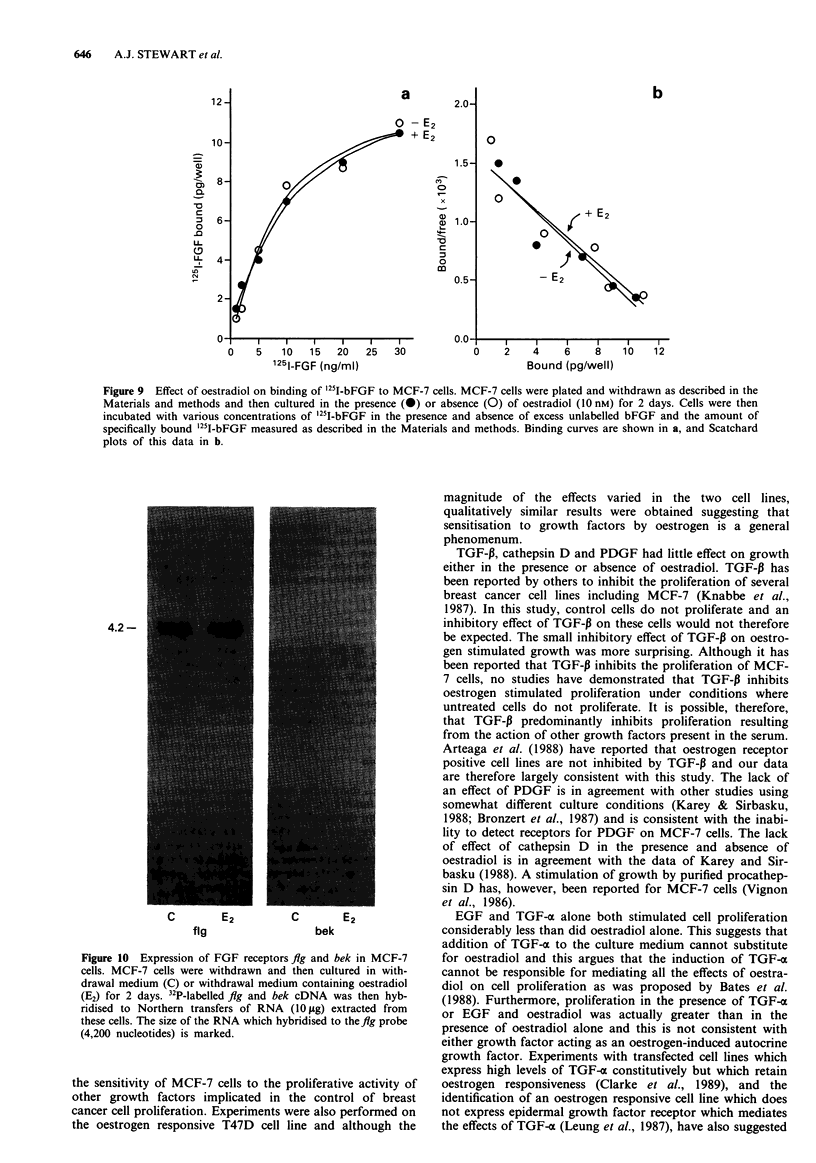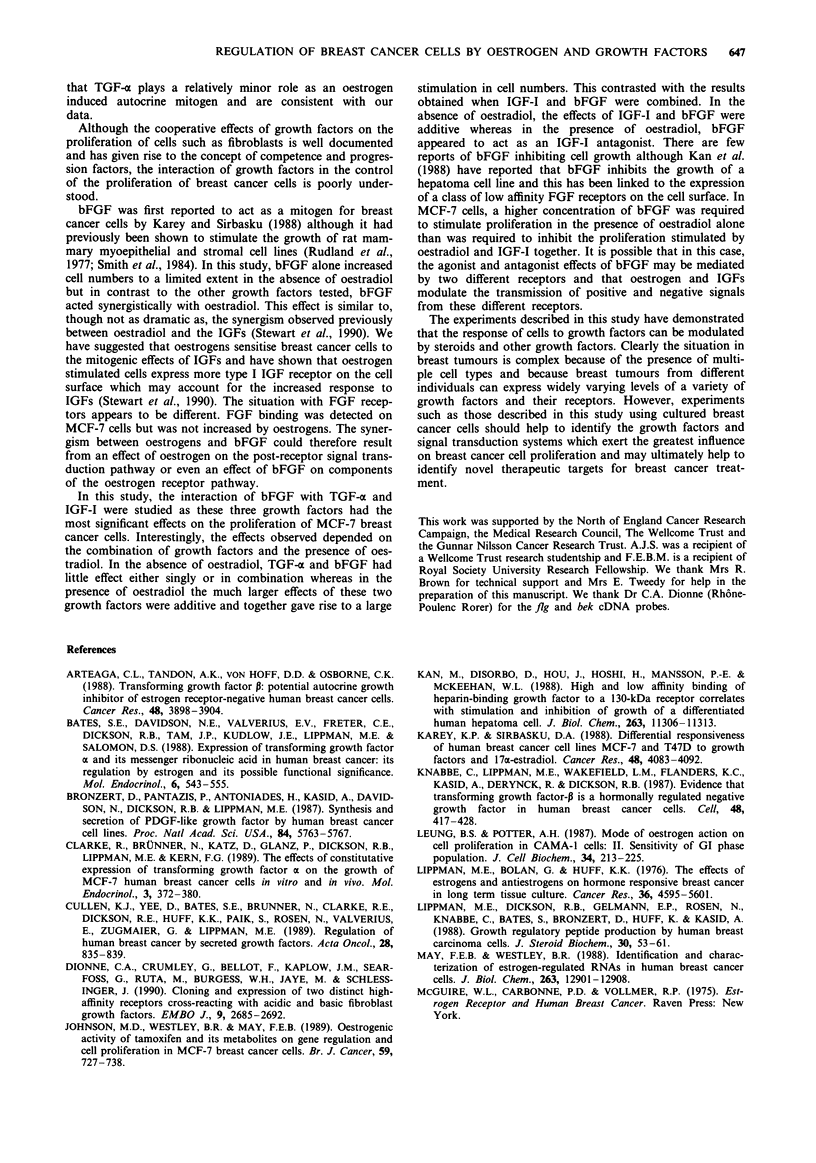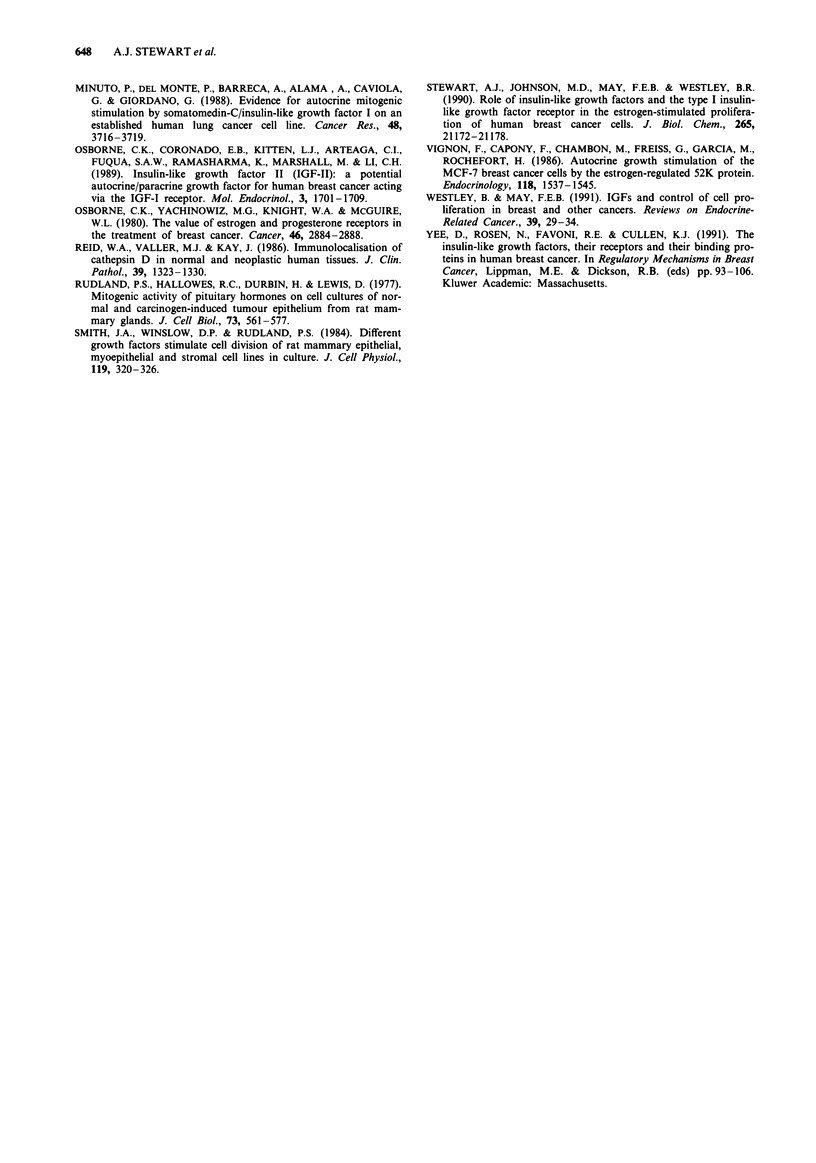# Modulation of the proliferative response of breast cancer cells to growth factors by oestrogen.

**DOI:** 10.1038/bjc.1992.330

**Published:** 1992-10

**Authors:** A. J. Stewart, B. R. Westley, F. E. May

**Affiliations:** Department of Pathology, Royal Victoria Infirmary, Newcastle upon Tyne, UK.

## Abstract

**Images:**


*Br. J. Cancer* (1992), **66**, 640–648 © Macmillan Press Ltd., 1992

### Modulation of the proliferative response of breast cancer cells to growth factors by oestrogen

A.J. Stewart, B.R. Westley & F.E.B. May

*Department of Pathology, Royal Victoria Infirmary, Newcastle upon Tyne, NE1 4LP, UK.*

**Summary** A number of growth factors have been implicated in the control of the proliferation of breast cancer cells and some have been reported to mediate the proliferative effects of oestradiol. MCF-7 cells were treated with growth factors in the presence and absence of oestradiol. Oestradiol increased the response of cells to the proliferative effects of epidermal growth factor (EGF), transforming growth factor alpha (TGF-α) and basic fibroblast growth factor (bFGF). Platelet derived growth factor (PDGF) and cathepsin D had no effect in the presence or absence of oestradiol while TGF-β slightly reduced the stimulation by oestradiol. In the absence of oestradiol, there was little effect of combinations of growth factors although the effects of bFGF and IGF-I were additive. In the presence of oestradiol, the effects of bFGF and TGF-α were additive whereas bFGF acted as an IGF-I antagonist. Overall, bFGF had the greatest effect on cell proliferation although this was less marked than the previously described effect of the IGFs and insulin. The effects of oestradiol on the sensitivity of cells to the proliferative effects of bFGF did not appear to result from regulation of bFGF receptor expression.

The factors which control the proliferation of breast cancer cells are not well defined. Breast tumours express variable levels of receptors for steroids (McGuire *et al.*, 1975; Osborne *et al.*, 1980) and growth factors (Cullen *et al.*, 1989) and it has been proposed that the ligands for these receptors act through a variety of autocrine and paracrine mechanisms. Studies *in vivo* and in cell culture systems have implicated a number of steroids and polypeptide growth factors (Lippman *et al.*, 1988) but the relative contribution of each growth factor and the precise way in which each acts is not known.

Breast cancer cell lines provide tractable experimental systems for examining some aspects of the control of breast cancer cell proliferation. Furthermore some cell lines such as the MCF-7 cell line contain oestrogen receptors and their proliferation is regulated by oestrogens (Lippman *et al.*, 1976; Johnson *et al.*, 1989). They therefore provide useful systems for studying the interactions between various growth factors and for understanding the mechanisms involved in the stimulation of proliferation by oestrogens and the interactions between growth factors and steroids.

Transforming growth factors (TGFs), insulin-like growth factors (IGFs), fibroblast growth factors (FGFs), platelet derived growth factor (PDGF) and epidermal growth factor (EGF) as well as other proteins such as cathepsin D have all been implicated in the control of breast cancer cell proliferation. Some of these growth factors, such as PDGF, are thought to be synthesised by tumour cells but to influence breast cancer cell proliferation indirectly by regulating the synthesis of other growth factors in stromal cells (Bronzert *et al.*, 1987). Other proteins, such as the transforming growth factors (Bates *et al.*, 1988; Knabbe *et al.*, 1987) and cathepsin D (Vignon *et al.*, 1986) have been reported to be synthesised by tumour cells and may act as autocrine mitogens. Regulation of the synthesis of autocrine growth factors such as TGF-α (Bates *et al.*, 1988) and TGF-β (Knabbe *et al.*, 1987) by oestrogens and antioestrogens has been reported and may contribute to mediating the effects of oestrogens on breast cancer cell proliferation.

The insulin-like growth factors are increasingly being implicated in the control of the proliferation of a wide variety of tumour cells (reviewed in Westley and May, 1991) and these proliferative effects are generally thought to be mediated through the type I IGF receptor. In some instances it has been proposed that tumour cells respond to exogenous IGFs, whereas in others such as small cell lung carcinoma, there is well documented evidence that IGF-I functions as an autocrine growth factor (Minuto *et al.*, 1988). In breast cancer, there is now a concensus that the cancer cells do not produce biologically significant amounts of IGF-I (Yee *et al.*, 1991) although some appear to produce IGF-II (Osborne *et al.*, 1989). We have reported that oestrogens sensitise breast cancer cells to the proliferative effects of IGFs (Stewart *et al.*, 1990) demonstrating that steroids can modulate the response of breast cancer cells to growth factors. In this study we have examined the effects of oestrogens on the response of breast cancer cells to other growth factors. We conclude that modulation of sensitivity does occur for other growth factors but that the mechanisms involved may differ from those involved for IGFs.

Correspondence: F.E.B. May.
Received 26 November 1991; accepted in revised form 15 June 1992.

### Materials and methods

### Materials

Recombinant IGF-I and TGFα were obtained from Bachem (UK). PDGF, bFGF and TGF-β1 were obtained from British Biotechnology. EGF was obtained from Boehringer Mannheim. Bovine insulin was obtained from Collaborative Research. Cathepsin D was purified from human spleen as described previously (Reid *et al.*, 1986).

### Cell culture

MCF-7 cells were maintained in Dulbecco's Modified Eagles medium supplemented with foetal calf serum (10%) and porcine insulin (1 $\mu g\ ml^{-1}$).

Growth experiments were performed as described previously (Johnson *et al.*, 1989). Cells (10,000) were plated in 16-mm diameter wells in 0.5 ml and allowed to attach over 2 days. Prior to treatment, the cells were progressively withdrawn from the oestrogens present in the normal growth medium as described by Johnson *et al.* (1989). This involved culturing the cells in phenol red-free modified Eagle's medium supplemented with charcoal-treated newborn calf serum (10%) and insulin (1 $\mu g\ ml^{-1}$) for 4 days. The medium was changed twice daily for the first 3 days with a PBS wash at every change. The cells were then cultured for a further 6 or 9 days in this medium which was supplemented with oestradiol and various combinations of growth factors. Culture medium was changed daily.

### *DNA assay*

DNA was measured using bisbenzimidazole (Hoechst 33258) as described previously (Johnson *et al.*, 1989).

### *FGF binding*

FGF binding was measured essentially as described by Kan *et al.* (1988). MCF-7 cells (20,000) were plated into 16 mm wells in normal growth medium. When the cells were 40% confluent, they were withdrawn from oestrogens present in normal growth medium as described for the growth experiments and then cells were treated with 10 nM oestradiol for 2 days or cultured for a further 2 days in withdrawal medium alone. Cell monolayers were washed with phosphate-buffered saline containing 1 mg $ml^{-1}$ BSA (PBS/BSA) and then incubated with varying concentrations of $^{125}$I-bFGF (1,400 Ci $mmol^{-1}$, Amersham, UK) in the presence and absence of a 100 fold molar excess of unlabelled bFGF in binding buffer (PBS/BSA supplemented with 2 $\mu$g $ml^{-1}$ heparin) for 2 h on ice. The cells were then washed four times with 0.5 ml of binding buffer and the bound $^{125}$I-bFGF was then extracted by incubation with 1% triton X-100 for 15 min on ice. The $^{125}$I-bFGF in the extraction buffer was measured in a gamma counter. Total and non-specific binding was measured in triplicate.

### *cDNA hybridisation*

Plasmids pDC115 and pCD116 containing cDNA corresponding to the extracellular domains of the *flg* and *bek* FGF receptors respectively (Dionne *et al.*, 1990) were labelled with $^{32}$P-dCTP by random priming. MCF-7 cells were withdrawn from oestrogens, and then treated for two days with oestradiol (10 nM) for 2 days as described above. Total RNA was extracted from control and oestrogen treated cells, electrophoresed on agarose gels containing 2.2 M formaldehyde and transferred to nylon filters. The radiolabelled *flg* and *bek* cDNA was then hybridised to the Northern transfers of RNA for 72 h at 42°C as described by May and Westley (1988).

All experiments were performed at least three times. In general, each point is the mean of three measurements and the bars represent the standard error of the mean.

### Results

### *Modulation of responsiveness of MCF-7 cells to growth factors by oestradiol*

In the first series of experiments, growth factors which have been implicated in the control of breast cancer cell proliferation were tested for their ability to stimulate the proliferation of MCF-7 cells in the absence and presence of oestradiol.

Prior to treatment, cells were cultured in phenol red-free medium containing charcoal-stripped serum. This medium has been shown previously to contain extremely low levels of oestrogen (May & Westley, 1988). After 5 days of culture in this medium, cells stop proliferating thus permitting small effects of added growth factors to be detected.

Under these culture conditions, oestradiol treatment routinely resulted in a two-four fold increase in cell numbers over 9 days. Addition of TGF-α or EGF alone (Figure 1a and b) resulted in a small, but statistically significant increase in cell numbers after 6 and 9 days of treatment. When cells were treated with TGF-α and oestradiol (Figure 1a) or EGF and oestradiol (Figure 1b), the increase in cell numbers was slightly greater than the sum of the increase observed in the presence of oestradiol alone and the growth factor alone. The increase in growth over 9 days was then measured at different concentrations of TGF-α (Figure 2a) and EGF (Figure 2b). The half-maximal increase was observed at 3 ng $ml^{-1}$ for TGF-α in both the presence and absence of oestradiol. In the

**Figure 1** Stimulation of MCF-7 cell proliferation by TGF-α **a**, and EGF **b**, in the presence and absence of oestradiol. MCF-7 cells were plated and withdrawn as described in the Materials and methods. In **a**, cells were cultured in withdrawal medium alone (O, C), withdrawal medium supplemented with TGF-α (10 ng $ml^{-1}$, ●), oestradiol (10 nM, □ $E_2$), or oestradiol and TGF-α together (■, TGF-α + $E_2$. In **b**, cells were cultured in withdrawal medium alone (O, C), withdrawal medium supplemented with EGF (10 ng $ml^{-1}$), oestradiol alone (□, $E_2$) or oestradiol and EGF together (■, EGF + $E_2$). The number of cells was measured after 6 and 9 days and expressedc as a percentage of the number of cells in wells treated with oestradiol.

**Figure 2** Dose response of the stimulation of MCF-7 cell proliferation by TGF-α **a**, and EGF **b**, in the presence and absence of oestradiol. MCF-7 cells were plated and withdrawn as described in the Materials and methods. They were then cultured for 9 days in withdrawal medium containing various concentrations of TGF-α **a**, or EGF **b**, in the presence (●) or absence (○) of oestradiol (10 nM).

**Figure 3** Effect of cathepsin D **a**, and PDGF **b**, on MCF-7 cell proliferation in the presence and absence of oestradiol. MCF-7 cells were plated and withdrawn as described in the Materials and methods. In **a**, cells were cultured in withdrawal medium alone (○, C), withdrawal medium supplemented with cathepsin D (5 ng ml$^{-1}$, ●, CD), oestradiol (10 nm, □, $E_2$) or cathepsin D and oestradiol together (■, CD + $E_2$). In **b**, cells were cultured in withdrawal medium alone (○, C), withdrawal medium supplemented with PDGF (10 ng ml$^{-1}$, ●, PDGF), oestradiol alone (10 nm, □, $E_2$) or oestradiol together with PDGF (■, PDGF + $E_2$). Cell numbers were measured as described in the legend to Figure 1.

presence of oestradiol, TGF-α increased cell proliferation 3 fold at concentrations of 10 μg ml$^{-1}$ and above but only 1.2 fold in the absence of oestradiol. For EGF half-maximal stimulation was observed at a somewhat lower concentration (1 ng ml$^{-1}$) but the maximal stimulation was similar to that observed for TGF-α.

Pro-cathepsin D has been reported to be mitogenic for MGF-7 cells (Vignon *et al.*, 1986). To test the mitogenic activity of mature cathepsin D, MCF-7 cells were treated with mature human cathepsin D purified from spleen. Figure 3a shows that there was no effect of cathepsin D (5 ng ml$^{-1}$) on proliferation in the presence or absence of oestradiol and this was true for all concentrations tested (1, 5 and 50 ng ml$^{-1}$). PDGF is a motogen which acts as a competence factor for fibroblasts. The mitogenic activity of PDGF was measured on MCF-7 cells in the presence and absence of oestrogen. Figure 3b shows that PDGF had no effect on cell proliferation and did not modulate the proliferative effect of oestradiol at a concentration 10 ng ml$^{-1}$. PDGF was then tested at a higher concentration (100 ng ml$^{-1}$) but again had no effect on the proliferation of withdrawn or oestrogen-treated MCF-7 cells.

Figure 4a and b show similar experiments with TGF-β and bFGF respectively. TGF-β alone had no effect on cell proliferation but marginally inhibited the stimulation of cell proliferation induced by oestradiol. bFGF alone marginally increased cell proliferation (1.4 fold) but treatment of MCF-7 cells with bFGF and oestradiol resulted in a significantly greater stimulation (8.8 fold) of growth than obtained with oestradiol alone (5 fold).

*Effects of combinations of growth factors on MCF-7 cell proliferation in the presence and absence of oestradiol*

Few studies have examined the effects of combinations of growth factors on the proliferation of breast cancer cells and none have studied the modulation of the effects of combinations of growth factors by oestradiol.

The growth inhibitory effect of TGF-β on breast cancer cell proliferation (Knabbe *et al.*, 1987) has provoked much interest. We have previously reported that insulin markedly stimulates the proliferation of breast cancer cells in the presence of oestradiol and we therefore examined whether TGF-β could inhibit the large stimulation of proliferation when MCF-7 cells are treated with insulin and oestradiol. Figure 5 shows the dramatic synergism between oestradiol and insulin. In this experiment, the stimulation by oestradiol and insulin together was five fold that observed for oestradiol alone. As in the experiment shown in Figure 4, TGF-β marginally decreased oestrogen-induced proliferation but did not reduce the much higher level of proliferation of cells treated with insulin and oestradiol together.

Of the growth factors which stimulated the proliferation of MCF-7 cells, TGF-α, EGF and bFGF (Figures 1–4) had the most pronounced effects. TGF-α and EGF had similar effects on the proliferation of MCF-7 cells, are both thought to act through the EGF receptor and are therefore unlikely to have an additive effect. However, as TGF-α and bFGF interact with different receptors, the effects of TGF-α together with bFGF were measured in the absence and presence of oestradiol. In the absence of oestradiol, both growth factors had little effect either alone or in combination (Figure 6a). In the presence of oestradiol, however, treatment of cells with TGF-α and bFGF resulted in a greater stimulation of proliferation (8.5 fold) than with either growth factor alone (Figure 6b). This experiment emphasised the dependence of the activity of these two growth factors on the presence of oestradiol and showed that they could also have additive effects in the presence of oestradiol.

Finally, the interaction between IGF-I and FGF was measured in the absence and presence of oestradiol to determine if bFGF could increase the proliferation stimulated by IGF-I. In the absence of oestradiol, neither IGF-I nor bFGF significantly increased cell proliferation but IGF-I and bFGF increased cell numbers two-fold after 9 days of treatment (Figure 7a). In the presence of oestradiol, however, bFGF

**Figure 4** Effect of TGF-β **a**, and bFGF **b**, on MCF-7 cell proliferation in the presence and absence of oestradiol. MCF-7 cells were plated and withdrawn as described in the Materials and methods. In **a**, cells were cultured in withdrawal medium alone (O, C), withdrawal medium supplemented with TGF-β (10 ng ml$^{-1}$, ●, TGF β), oestradiol alone (10 nm, □, $E_2$). In **b**, cells were cultured in withdrawal medium alone (O, C), withdrawal medium supplemented with bFGF (10 ng ml$^{-1}$, ●, bFGF), oestradiol alone (10 nm, □, $E_2$) or oestradiol together with bFGF (■, bFGF + $E_2$). Cell numbers were measured as described in the legend to Figure 1.

inhibited the effects of IGF-I so that the number of cells after 9 days of treatment was similar to that following treatment with bFGF in the presence of oestradiol (Figure 7b).

**Figure 5** Effect of TGF-β on MCF-7 cell proliferation stimulated by oestradiol and insulin. MCF-7 cells were plated and withdrawn as described in Materials and methods. Cells were cultered in withdrawal medium alone **c**, withdrawal medium supplemented with oestradiol (10 nM, $E_2$), insulin (1 $\mu g\ ml^{-1}$) or oestradiol and insulin in the presence (solid) or absence (open) of TGF β (10 ng $ml^{-1}$). Cell numbers were measured as described in the legend to Figure 1.

The concentrations of bFGF which were synergistic with oestradiol and which inhibited the effect of IGF-I in the presence of oestradiol were defined by culturing MCF-7 cells in the presence of various concentrations of bFGF with oestradiol alone or IGF-I together with oestradiol (Figure 8). Both effects of bFGF were dose dependent and were maximal at 100 pg $ml^{-1}$. However, significant antagonist effects were observed at 1 and 10 pg $ml^{-1}$ whereas no agonist effects were observed at this concentration. Half maximal stimulation in the presence of oestradiol alone was observed at 30 pg while half maximal inhibition in the presence of oestradiol and IGF-I occurred at 7 pg bFGF.

*Characterisation of bFGF binding to MCF-7 cells*

bFGF had the clearest synergistic effect of the growth factors used in this study. We have previously reported that oestradiol increases expression of the type I IGF receptor on MCF-7 cells (Stewart *et al.*, 1990) and this may account for the synergism between oestradiol and the IGFs. The effect of oestradiol on bFGF binding was therefore measured. High affinity binding of bFGF was measured as described in the Materials and methods. Specific bFGF binding accounted for approximately 60% of total binding as judged by the suppression of $^{125}$I-bFGF binding by an excess (100 fold) of unlabelled bFGF. Figure 9a shows binding curves of $^{125}$I-bFGF to withdrawn and oestrogen treated MCF-7 cells and Figure 9b Scatchard plots of this data. The Scatchard plot is consistent with a single class of binding sites and there was no difference between the number of binding sites (9,600 sites/cell) or their affinity (0.6 nM) in withdrawn and oestrogen treated cells suggesting that expression of bFGF receptors is not regulated by oestrogen.

The expression of two FGF receptors (*flg* and *bek*) was then examined. cDNA corresponding to the extracellular

**Figure 6** Effect of oestradiol on MCF-7 cell proliferation stimulated by TGF-α and bFGF together. MCF-7 cells were plated and withdrawn as described in the Materials and methods. In **a**, cells were cultured in withdrawal medium alone (O, C), or withdrawal medium supplemented with TGF-α (10 ng $ml^{-1}$, Δ, TGF-α), bFGF (10 ng $ml^{-1}$, ∇, bFGF) or TGF-α and bFGF together (☆, bFGF + TGF-α). In **b**, cells were cultured in withdrawal medium supplemented with oestradiol (●, $E_2$ alone) or oestradiol together with TGE-α (10 ng $ml^{-1}$, ▲, + TGF-α), bFGF (10 ng $ml^{-1}$, ∇, + bFGF) or TGF-α and bFGF (★, + bFGF + TGF-α). Cell numbers were measured as described in the legend to Figure 1.

**Figure 7** Effect of oestradiol on MCF-7 cell proliferation stimulated by IGF-I and bFGF together. MCF-7 cells were plated and withdrawn as described in the Materials and methods. In **a**, cells were cultured in withdrawal medium alone (O, C), withdrawal medium supplemented with IGF-I (50 ng ml$^{-1}$, ▽, IGF-I), bFGF (10 ng ml$^{-1}$, △, bFGF) or IGF-I and bFGF together (☆, IGF-I + bFGF). In **b**, cells were cultured in withdrawal medium supplemented with oestradiol (10 nm ml$^{-1}$, ▲, + bFGF), IGF-I (50 ng ml$^{-1}$, ▼, + IGF-I) or bFGF and IGF-I (★, + IGF-I + bFGF). Cell numbers were measured as described in the legend to Figure 1.

**Figure 8** Effect of bFGF on the proliferation of MCF-7 cell proliferation stimulated by oestradiol and IGF-I. MCF-7 cells were plated and withdrawn as described in the Materials and methods. Cells were cultured for 9 days in withdrawal medium alone (unhatched), withdrawal medium supplemented with oestradiol alone (10 nM), hatched), withdrawal medium supplemented with oestradiol (10 nM) and the indicated concentration of bFGF (O) or withdrawal medium supplemented with oestradiol (10 nM), IGF-I (50 ng ml$^{-1}$) and the indicated concentration of bFGF (●). Cell numbers were measured as described in the legend to Figure 1.

domains of the two receptors was hybridised to Northern transfers of RNA extracted from withdrawn and oestrogen-treated MCF-7 cells. No hybridisation of the *bek* cDNA was detected, however the *flg* cDNA hybridised to an RNA of 4.2 kb but this RNA was expressed at the same level in withdrawn and oestrogen treated cells (Figure 10).

### Discussion

Although there have been extensive studies on the effects of growth factors on the proliferation of breast cancer cells as well as on the production of growth factors by breast cancer cells, there have been relatively few studies on the interaction between steroids and growth factors. Given that oestrogens are important in tumour development *in vivo* and that oestrogens may mediate their effects through growth factor pathways, this is an area of some importance.

The analysis of the effects of oestrogens on breast cancer cells has been facilitated by the development of oestrogen-free culture conditions and the withdrawal of cells from oestrogens present in normal culture media prior to treatment. This involves the use of phenol-red free medium supplemented with stripped serum and a withdrawal period which involves frequent changes of medium to remove the influences of steroids present in normal growth medium. These culture conditions have been used to identify oestrogen regulated mRNAs in breast cancer cells (May & Westley, 1988) and to study the effects of antioestrogens on the expression of oestrogen regulated genes (Johnson *et al.*, 1989).

We have previously reported that oestrogens sensitise three oestrogen responsive breast cancer cell lines to the proliferative effects of IGFs (Stewart *et al.*, 1990). In the present study we have examined the ability of oestrogen to modulate

**Figure 9** Effect of oestradiol on binding of $^{125}$I-bFGF to MCF-7 cells. MCF-7 cells were plated and withdrawn as described in the Materials and methods and then cultured in the presence (●) or absence (O) of oestradiol (10 nM) for 2 days. Cells were then incubated with various concentrations of $^{125}$I-bFGF in the presence and absence of excess unlabelled bFGF and the amount of specifically bound $^{125}$I-bFGF measured as described in the Materials and methods. Binding curves are shown in **a**, and Scatchard plots of this data in **b**.

**Figure 10** Expression of FGF receptors *flg* and *bek* in MCF-7 cells. MCF-7 cells were withdrawn and then cultured in withdrawal medium (C) or withdrawal medium containing oestradiol ($E_2$) for 2 days. $^{32}$P-labelled *flg* and *bek* cDNA was then hybridised to Northern transfers of RNA (10 μg) extracted from these cells. The size of the RNA which hybridised to the *flg* probe (4,200 nucleotides) is marked.

the sensitivity of MCF-7 cells to the proliferative activity of other growth factors implicated in the control of breast cancer cell proliferation. Experiments were also performed on the oestrogen responsive T47D cell line and although the magnitude of the effects varied in the two cell lines, qualitatively similar results were obtained suggesting that sensitisation to growth factors by oestrogen is a general phenomenum.

TGF-β, cathepsin D and PDGF had little effect on growth either in the presence or absence of oestradiol. TGF-β has been reported by others to inhibit the proliferation of several breast cancer cell lines including MCF-7 (Knabbe *et al.*, 1987). In this study, control cells do not proliferate and an inhibitory effect of TGF-β on these cells would not therefore be expected. The small inhibitory effect of TGF-β on oestrogen stimulated growth was more surprising. Although it has been reported that TGF-β inhibits the proliferation of MCF-7 cells, no studies have demonstrated that TGF-β inhibits oestrogen stimulated proliferation under conditions where untreated cells do not proliferate. It is possible, therefore, that TGF-β predominantly inhibits proliferation resulting from the action of other growth factors present in the serum. Arteaga *et al.* (1988) have reported that oestrogen receptor positive cell lines are not inhibited by TGF-β and our data are therefore largely consistent with this study. The lack of an effect of PDGF is in agreement with other studies using somewhat different culture conditions (Karey & Sirbasku, 1988; Bronzert *et al.*, 1987) and is consistent with the inability to detect receptors for PDGF on MCF-7 cells. The lack of effect of cathepsin D in the presence and absence of oestradiol is in agreement with the data of Karey and Sirbasku (1988). A stimulation of growth by purified procathepsin D has, however, been reported for MCF-7 cells (Vignon *et al.*, 1986).

EGF and TGF-α alone both stimulated cell proliferation considerably less than did oestradiol alone. This suggests that addition of TGF-α to the culture medium cannot substitute for oestradiol and this argues that the induction of TGF-α cannot be responsible for mediating all the effects of oestradiol on cell proliferation as was proposed by Bates *et al.* (1988). Furthermore, proliferation in the presence of TGF-α or EGF and oestradiol was actually greater than in the presence of oestradiol alone and this is not consistent with either growth factor acting as an oestrogen-induced autocrine growth factor. Experiments with transfected cell lines which express high levels of TGF-α constitutively but which retain oestrogen responsiveness (Clarke *et al.*, 1989), and the identification of an oestrogen responsive cell line which does not express epidermal growth factor receptor which mediates the effects of TGF-α (Leung *et al.*, 1987), have also suggested that TGF-α plays a relatively minor role as an oestrogen induced autocrine mitogen and are consistent with our data.

Although the cooperative effects of growth factors on the proliferation of cells such as fibroblasts is well documented and has given rise to the concept of competence and progression factors, the interaction of growth factors in the control of the proliferation of breast cancer cells is poorly understood.

bFGF was first reported to act as a mitogen for breast cancer cells by Karey and Sirbasku (1988) although it had previously been shown to stimulate the growth of rat mammary myoepithelial and stromal cell lines (Rudland *et al.*, 1977; Smith *et al.*, 1984). In this study, bFGF alone increased cell numbers to a limited extent in the absence of oestradiol but in contrast to the other growth factors tested, bFGF acted synergistically with oestradiol. This effect is similar to, though not as dramatic as, the synergism observed previously between oestradiol and the IGFs (Stewart *et al.*, 1990). We have suggested that oestrogens sensitise breast cancer cells to the mitogenic effects of IGFs and have shown that oestrogen stimulated cells express more type I IGF receptor on the cell surface which may account for the increased response to IGFs (Stewart *et al.*, 1990). The situation with FGF receptors appears to be different. FGF binding was detected on MCF-7 cells but was not increased by oestrogens. The synergism between oestrogens and bFGF could therefore result from an effect of oestrogen on the post-receptor signal transduction pathway or even an effect of bFGF on components of the oestrogen receptor pathway.

In this study, the interaction of bFGF with TGF-α and IGF-I were studied as these three growth factors had the most significant effects on the proliferation of MCF-7 breast cancer cells. Interestingly, the effects observed depended on the combination of growth factors and the presence of oestradiol. In the absence of oestradiol, TGF-α and bFGF had little effect either singly or in combination whereas in the presence of oestradiol the much larger effects of these two growth factors were additive and together gave rise to a large stimulation in cell numbers. This contrasted with the results obtained when IGF-I and bFGF were combined. In the absence of oestradiol, the effects of IGF-I and bFGF were additive whereas in the presence of oestradiol, bFGF appeared to act as an IGF-I antagonist. There are few reports of bFGF inhibiting cell growth although Kan *et al.* (1988) have reported that bFGF inhibits the growth of a hepatoma cell line and this has been linked to the expression of a class of low affinity FGF receptors on the cell surface. In MCF-7 cells, a higher concentration of bFGF was required to stimulate proliferation in the presence of oestradiol alone than was required to inhibit the proliferation stimulated by oestradiol and IGF-I together. It is possible that in this case, the agonist and antagonist effects of bFGF may be mediated by two different receptors and that oestrogen and IGFs modulate the transmission of positive and negative signals from these different receptors.

The experiments described in this study have demonstrated that the response of cells to growth factors can be modulated by steroids and other growth factors. Clearly the situation in breast tumours is complex because of the presence of multiple cell types and because breast tumours from different individuals can express widely varying levels of a variety of growth factors and their receptors. However, experiments such as those described in this study using cultured breast cancer cells should help to identify the growth factors and signal transduction systems which exert the greatest influence on breast cancer cell proliferation and may ultimately help to identify novel therapeutic targets for breast cancer treatment.

This work was supported by the North of England Cancer Research Campaign, the Medical Research Council, The Wellcome Trust and the Gunnar Nilsson Cancer Research Trust. A.J.S. was a recipient of a Wellcome Trust research studentship and F.E.B.M. is a recipient of Royal Society University Research Fellowship. We thank Mrs R. Brown for technical support and Mrs E. Tweedy for help in the preparation of this manuscript. We thank Dr C.A. Dionne (Rhône-Poulenc Rorer) for the *flg* and *bek* cDNA probes.

### References

ARTEAGA, C.L., TANDON, A.K., VON HOFF, D.D. & OSBORNE, C.K. (1988). Transforming growth factor β: potential autocrine growth inhibitor of estrogen receptor-negative human breast cancer cells. *Cancer Res.*, **48**, 3898–3904.

BATES, S.E., DAVIDSON, N.E., VALVERIUS, E.V., FRETER, C.E., DICKSON, R.B., TAM, J.P., KUDLOW, J.E., LIPPMAN, M.E. & SALOMON, D.S. (1988). Expression of transforming growth factor α and its messenger ribonucleic acid in human breast cancer: its regulation by estrogen and its possible functional significance. *Mol. Endocrinol.*, **6**, 543–555.

BRONZERT, D., PANTAZIS, P., ANTONIADES, H., KASID, A., DAVIDSON, N., DICKSON, R.B. & LIPPMAN, M.E. (1987). Synthesis and secretion of PDGF-like growth factor by human breast cancer cell lines. *Proc. Natl Acad. Sci. USA.*, **84**, 5763–5767.

CLARKE, R., BRÜNNER, N., KATZ, D., GLANZ, P., DICKSON, R.B., LIPPMAN, M.E. & KERN, F.G. (1989). The effects of constitutative expression of transforming growth factor α on the growth of MCF-7 human breast cancer cells *in vitro* and *in vivo*. *Mol. Endocrinol.*, **3**, 372–380.

CULLEN, K.J., YEE, D., BATES, S.E., BRUNNER, N., CLARKE, R.E., DICKSON, R.E., HUFF, K.K., PAIK, S., ROSEN, N., VALVERIUS, E., ZUGMAIER, G. & LIPPMAN, M.E. (1989). Regulation of human breast cancer by secreted growth factors. *Acta Oncol.*, **28**, 835–839.

DIONNE, C.A., CRUMLEY, G., BELLOT, F., KAPLOW, J.M., SEARFOSS, G., RUTA, M., BURGESS, W.H., JAYE, M. & SCHLESSINGER, J. (1990). Cloning and expression of two distinct high-affinity receptors cross-reacting with acidic and basic fibroblast growth factors. *EMBO J.*, **9**, 2685–2692.

JOHNSON, M.D., WESTLEY, B.R. & MAY, F.E.B. (1989). Oestrogenic activity of tamoxifen and its metabolites on gene regulation and cell proliferation in MCF-7 breast cancer cells. *Br. J. Cancer*, **59**, 727–738.

KAN, M., DISORBO, D., HOU, J., HOSHI, H., MANSSON, P.-E. & MCKEEHAN, W.L. (1988). High and low affinity binding of heparin-binding growth factor to a 130-kDa receptor correlates with stimulation and inhibition of growth of a differentiated human hepatoma cell. *J. Biol. Chem.*, **263**, 11306–11313.

KAREY, K.P. & SIRBASKU, D.A. (1988). Differential responsiveness of human breast cancer cell lines MCF-7 and T47D to growth factors and 17α-estradiol. *Cancer Res.*, **48**, 4083–4092.

KNABBE, C., LIPPMAN, M.E., WAKEFIELD, L.M., FLANDERS, K.C., KASID, A., DERYNCK, R. & DICKSON, R.B. (1987). Evidence that transforming growth factor-β is a hormonally regulated negative growth factor in human breast cancer cells. *Cell*, **48**, 417–428.

LEUNG, B.S. & POTTER, A.H. (1987). Mode of oestrogen action on cell proliferation in CAMA-1 cells: II. Sensitivity of GI phase population. *J. Cell Biochem.*, **34**, 213–225.

LIPPMAN, M.E., BOLAN, G. & HUFF, K.K. (1976). The effects of estrogens and antiestrogens on hormone responsive breast cancer in longterm tissue culture. *Cancer Res.*, **36**, 4595–5601.

LIPPMAN, M.E., DICKSON, R.B., GELMANN, E.P., ROSEN, N., KNABBE, C., BATES, S., BRONZERT, D., HUFF, K. & KASID, A. (1988). Growth regulatory peptide production by human breast carcinoma cells. *J. Steroid Biochem.*, **30**, 53–61.

MAY, F.E.B. & WESTLEY, B.R. (1988). Identification and characterization of estrogen-regulated RNAs in human breast cancer cells. *J. Biol. Chem.*, **263**, 12901–12908.

MCGUIRE, W.L., CARBONNE, P.D. & VOLLMER, R.P. (1975). *Estrogen Receptor and Human Breast Cancer*. Raven Press: New York.

MINUTO, P., DEL MONTE, P., BARRECA, A., ALAMA , A., CAVIOLA, G. & GIORDANO, G. (1988). Evidence for autocrine mitogenic stimulation by somatomedin-C/insulin-like growth factor I on an established human lung cancer cell line. *Cancer Res.*, **48**, 3716-3719.

OSBORNE, C.K., CORONADO, E.B., KITTEN, L.J., ARTEAGA, C.I., FUQUA, S.A.W., RAMASHARMA, K., MARSHALL, M. & LI, C.H. (1989). Insulin-like growth factor II (IGF-II): a potential autocrine/paracrine growth factor for human breast cancer acting via the IGF-I receptor. *Mol. Endocrinol.*, **3**, 1701-1709.

OSBORNE, C.K., YACHINOWIZ, M.G., KNIGHT, W.A. & MCGUIRE, W.L. (1980). The value of estrogen and progesterone receptors in the treatment of breast cancer. *Cancer*, **46**, 2884-2888.

REID, W.A., VALLER, M.J. & KAY, J. (1986). Immunolocalisation of cathepsin D in normal and neoplastic human tissues. *J. Clin. Pathol.*, **39**, 1323-1330.

RUDLAND, P.S., HALLOWES, R.C., DURBIN, H. & LEWIS, D. (1977). Mitogenic activity of pituitary hormones on cell cultures of normal and carcinogen-induced tumour epithelium from rat mammary glands. *J. Cell Biol.*, **73**, 561-577.

SMITH, J.A., WINSLOW, D.P. & RUDLAND, P.S. (1984). Different growth factors stimulate cell division of rat mammary epithelial, myoepithelial and stromal cell lines in culture. *J. Cell Physiol.*, **119**, 320-326.

STEWART, A.J., JOHNSON, M.D., MAY, F.E.B. & WESTLEY, B.R. (1990). Role of insulin-like growth factors and the type I insulin-like growth factor receptor in the estrogen-stimulated proliferation of human breast cancer cells. *J. Biol. Chem.*, **265**, 21172-21178.

VIGNON, F., CAPONY, F., CHAMBON, M., FREISS, G., GARCIA, M., ROCHEFORT, H. (1986). Autocrine growth stimulation of the MCF-7 breast cancer cells by the estrogen-regulated 52K protein. *Endocrinology*, **118**, 1537-1545.

WESTLEY, B. & MAY, F.E.B. (1991). IGFs and control of cell proliferation in breast and other cancers. *Reviews on Endocrine-Related Cancer.*, **39**, 29-34.

YEE, D., ROSEN, N., FAVONI, R.E. & CULLEN, K.J. (1991). The insulin-like growth factors, their receptors and their binding proteins in human breast cancer. In *Regulatory Mechanisms in Breast Cancer*, Lippman, M.E. & Dickson, R.B. (eds) pp. 93-106. Kluwer Academic: Massachusetts.